# Assessing *L. donovani* Skin Parasite Load: A Proof of Concept Study of a Microbiopsy Device in an Indian Setting

**DOI:** 10.3389/fcimb.2021.645121

**Published:** 2021-03-11

**Authors:** Kristien Cloots, Om Prakash Singh, Abhishek Kumar Singh, Gert Van der Auwera, Prashant Kumar, Mallikarjuna Rao Gedda, Tulika Kumari Rai, Epco Hasker, Shyam Sundar, Marleen Boelaert

**Affiliations:** ^1^Department of Public Health, Institute of Tropical Medicine, Antwerp, Belgium; ^2^Department of Biochemistry, Institute of Science, Banaras Hindu University, Varanasi, India; ^3^Infectious Diseases Research Laboratory, Department of Medicine, Institute of Medical Sciences, Banaras Hindu University, Varanasi, India; ^4^Department of Biomedical Sciences, Institute of Tropical Medicine, Antwerp, Belgium; ^5^Kala-azar Medical Research Centre, Muzaffarpur, India

**Keywords:** Leishmaniasis, skin parasites, infectiousness, innovative tools, India, proof of concept study

## Abstract

**Background:**

In the endgame of the elimination initiative of visceral leishmaniasis (VL) on the Indian subcontinent, one of the main questions remaining is whether asymptomatically infected individuals also contribute to transmission. We piloted a minimally invasive microbiopsy device that could help answer this question. While the potential of this device has been previously illustrated in Ethiopia, no such information is available for the setting of the Indian subcontinent. In this proof of concept study we aimed to assess 1) to what extent skin parasite load obtained with the new microbiopsy device correlates with disease status, 2) to what extent skin parasite load correlates with blood parasite load in the same subject, and 3) to what extent the skin parasite load obtained from different sampling sites on the body correlates with one another.

**Methods:**

We performed a pilot study in Bihar, India, including 29 VL patients, 28 PKDL patients, 94 asymptomatically infected individuals, 22 endemic controls (EC), and 28 non-endemic controls (NEC). Presence of infection with *L. donovani* in the blood was assessed using Direct Agglutination Test, rK39 ELISA, Whole Blood Analysis measuring IFN-γ and qPCR. A skin sample was collected with the microbiopsy device on two different locations on the body. PKDL patients provided a third skin sample from the edge of a PKDL lesion. Parasite load in the skin was measured by qPCR.

**Findings:**

We found a clear correlation between the skin parasite load obtained with the microbiopsy device and disease status, with both higher skin parasite loads and higher proportions of positive skin samples in VL and PKDL patients compared to asymptomatics, EC, and NEC. No clear correlation between skin parasite load and blood parasite load was found, but a moderate correlation was present between the skin parasite load in arm and neck samples. In addition, we found four positive skin samples among asymptomatic individuals, and 85% of PKDL lesions tested positive using this microbiopsy device.

**Conclusions:**

In line with previous pilot studies, our results from an Indian setting suggest that the microbiopsy device provides a promising tool to measure skin parasite load, and – if validated by xenodiagnosis studies – could facilitate much needed larger scale studies on infectiousness of human subgroups. In addition, we advocate further evaluation of this device as a diagnostic tool for PKDL.

## Introduction

Visceral Leishmaniasis (VL), caused by the parasite *Leishmania donovani*, is a Neglected Tropical Disease which is currently on the verge of being eliminated as a public health problem (target annual incidence <1/10,000 population on (sub-)district level) in the Indian subcontinent (World Health Organization ROfS-EA, 2005). In this endgame, however, new challenges arise, the main question being whether elimination of transmission – in contrast to elimination of the disease - in the region is technically feasible.

One of the main issues remaining is whether asymptomatically infected individuals contribute to the spreading of this disease. Asymptomatically infected individuals are those with positive immunological or parasitological markers of *L. donovani* while not showing any signs or symptoms of VL. The currently available tools, however, do not allow large scale studies on this topic. The golden standard for assessing infectiousness is xenodiagnosis, in which laboratory-bred sand flies feed upon a suspectedly infected individual and are examined for infection some hours to days later. This is a very cumbersome method, with major practical, budgetary and ethical constraints that limit its use in humans to small scale, highly controlled laboratory studies (Singh et al., 2020a). Therefore parasite load in the blood, measured by qPCR, is often used as a proxy for infectiousness in humans. Several studies, however, suggest that skin parasite load is a better proxy for infectiousness to sand flies than blood parasite load (Courtenay et al., 2014; Doehl et al., 2017). This is not unexpected, because sand flies are telmophages, meaning they feed on blood pools they create by lacerating the sskin. Normal skin biopsies, however, are too painful and ethically unacceptable to be used on a large scale to survey asymptomatic individuals.

Recently, Lin et al. developed a microbiopsy (MB) device that may provide an alternative for a standard skin biopsy (Lin et al., 2013). This device takes minimally invasive and virtually painless skin samples that mimic the amount and the composition of the tissue ingested by a sand fly. Two studies performed in Ethiopia have illustrated the potential of this MB tool to take up *Leishmania* DNA (*L. ethiopica* and *L. donovani*) in both patients and a part of asymptomatically infected individuals (Kirstein et al., 2017; Churiso et al., 2020). We set out to pilot this microbiopsy device in an Indian setting. In this proof of concept (phase 1) study, we aimed to evaluate this new diagnostic tool on different population subsets based on disease status and compare the outcomes of the parasite skin load as measured with this MB device with parasitemia as measured with standard molecular tools. The objectives of this study were to assess 1) to what extent skin parasite load obtained with the new microbiopsy device correlates with disease status (symptomatic versus asymptomatic, PKDL and non-endemic subjects), 2) to what extent skin parasite load correlates with blood parasite load in the same subject, and 3) to what extent the skin parasite loads obtained from different sampling sites on the body correlate with one another.

## Materials and Method*s*

### Study Site and Participants

Recruitment of participants took place between August 2018 and January 2019. VL and PKDL patients were recruited at the Kala-Azar Medical Research Center (KAMRC) in Muzaffarpur, Bihar, India. VL and PKDL diagnosis were confirmed as per hospital guidelines, using clinical presentation in combination with a positive rK39 Rapid Diagnostic Test (RDT) for VL, and clinical presentation in combination with either microscopy or PCR on a skin slit smear for the diagnosis of PKDL. All patients were tested for HIV, and VL-HIV coinfected patients were excluded from this study.

A blood sample was taken from residents of highly endemic VL villages within Bihar state, and tested for signs of infection, using rK39 ELISA, Direct Agglutination Test (DAT), Whole Blood Analysis (WBA), and qPCR (details on all tests are provided below). Individuals positive on any of these tests were categorized as asymptomatically infected with *L. donovani*. In absence of any positive test, individuals were categorized as endemic controls.

Students residing at Banaras Hindu University (BHU) in Varanasi, Uttar Pradesh, India, were approached as potential non-endemic controls. They provided a 5 ml blood sample to be tested for *L. donovani* infection (using the same four tests, namely rK39 ELISA, DAT, WBA, and qPCR), and were excluded in case of any positive finding. Additional exclusion criteria for non-endemic controls were a) any episode of fever in the last month, b) skin lesions resembling PKDL, c) previous episode of VL or PKDL in the participant or a household member, and d) overnight stay in the last six months in a VL endemic area.

Skin samples were taken from all participants from two different sampling sites which are considered to be among the preferred spots for sand fly bites; the lower arm and the nape of the neck. For PKDL patients one additional sample was taken from the edge of one of the PKDL lesions. The time interval in between blood and skin sampling was no more than 14 days (for DAT and rK39 ELISA), 2 months (for WBA), and 1 h (for qPCR). Laboratory results were entered on pre-printed paper sheets, using barcodes to ensure participant protection during analysis. Data underwent double data entry in an Access database at BHU. Data analysis and graphical presentation was performed with R Studio version 3.6.2.

### Microbiopsy Devices and Skin Sampling

The microbiopsy device (MB) used was a single use prototype device [Trajan Scientific and Medical, Ringwood, Australia, described by Lin et al. (2013)]. It was developed to mimic the bite of a sand fly, penetrating the skin to the level of the dermis (200 µm) and taking up both blood and skin tissue. The tip of the device consists of two pointed outer plates and a central bifurcated one, creating a chamber volumetric size of 0.003 mm^3^ for sampling.

The skin of the participant was disinfected with 70% alcohol, after which the spring-loaded tip of the sampling device was released, while continuously pressing it against the skin for 30 s. Presence of blood was confirmed by the eye to ensure that a sample was taken. If blood was present, the microblade containing the sample was removed from the spring device with a sterile forceps, and stored in a dry sterile tube at −80°C until further analysis. All samples from symptomatic cases were taken prior to receiving any treatment.

### Laboratory Tests

#### Leishmania Infection in Blood

For the detection of antibodies against *L. donovani* we used DAT and rK39 ELISA. DAT was carried out using a kit from the Institute of Tropical Medicine, Antwerp, as described elsewhere (Jacquet et al., 2006). A cut-off titer ≥ 1:1,600 was used to define infection (Hasker et al., 2013). rK39 ELISA was performed as described previously, using the same cut-off titer of ≥ 14 percentage point positivity (pp) to define a positive test as identified in the paper of Hasker et al. (mean value for a healthy non-endemic control plus three standard deviations) (Hasker et al., 2013). Whole Blood Analysis (WBA) was performed as described elsewhere (Singh et al., 2021). In short, antigen-specific IFN-γ levels produced in response to soluble *Leishmania* antigen (SLA) stimulation were determined by subtracting background levels measured in the non-stimulated samples. The result was considered positive when the IFN-γ concentration in the antigen wells was 52.28 pg/ml or higher (Singh et al., 2021).

#### Parasite Load in Blood

Blood samples for determining parasite load in the blood (through qPCR) were taken within 1 h of the skin samples to assure maximum correlation in time with the parasite load in the skin samples. DNA was extracted using the QIAamp DNA blood mini kit according to the instructions of the manufacturer (www.qiagen.com). DNA samples were eluted in 80 µl of AE elution buffer. *Leishmania* kinetoplast minicircle DNA was amplified by the TaqMan-based qPCR assay. It was conducted in duplicate in a final 20 µl volume comprising of 10 µl TaqMan^®^ Universal Master Mix II with UNG (Applied Biosystems, Carlsbad, CA, USA), 4 µl DNA template, 0.1 mg/ml of Acetylated BSA (AcBSA-Promega), 0.6 µM of each primer, and 0.4 µM of FAM labeled probe (Integrated DNA Technologies, Coralville, IA, USA) from Mary et al. (2004). PCR and cycling conditions were 2 min at 50°C for UNG incubation and 10 min at 95°C and 40 cycles at 95°C for 15 s and 60°C for 1 min. The DNA was quantified by comparison to a standard curve derived from a microscopically quantified promastigote culture (*L. donovani* LEM 138), ranging from 1,000 down to 0.01 parasite genomes per PCR, in steps of 10. The limit of the linear dynamic range was 3.75 PGE per ml blood, whereby 1 PGE (parasite genome equivalent) corresponds to the amount of kDNA minicircles in a single parasite. Quantifying PGE/ml was only possible when surpassing the threshold of the linear dynamic range; values below this threshold were scored the average value of the unquantifiable range (1.875 PGE/ml) for further analysis, or 0 PGE/ml if no PCR amplicon was detected. The cut-off value for defining positivity was set at 37.5 PGE/ml blood, corresponding to one PGE per PCR, and was higher than the maximum value in any technical (no-template) control in order to avoid false positives.

#### Parasite Load in Skin

DNA was extracted from the microblades (MB) using the QIAamp DNA micro kit in accordance with the manufacturer’s instructions (www.qiagen.com). Per MB, 1 µg carrier RNA was added, and DNA was eluted in 20 µl buffer AE, both provided by the manufacturer as part of the kit. Amplification and quantification of *Leishmania* kDNA minicircles was performed on the same way as described for blood samples. The cut-off value to define positivity was set at one PGE per PCR, corresponding to 5 PGE per MB. This cut-off was higher than the maximum value in any technical negative (no-template) control in order to avoid false positives. Quantification was possible only down to 0.5 PGE/MB (limit of the linear dynamic range); values below this threshold (< 0.5 PGE/MB) were scored the average value of the unquantifiable range (0.25 PGE/MB), or negative when no PCR amplicon was detected.

To evaluate the quantity of human DNA in all samples, amplification of the RNase P gene (Applied Biosystems, Catalogue number: 4403326) was used as an internal reference of genomic human DNA. Four microliters of extracted DNA was added to 20 μl of PCR mastermix and amplification was performed on the same ABI 7500 Real-Time PCR machine [Applied Biosystems (ABI), Carlsbad, CA, USA]. Quantification of the RNase P gene copies present in each sample was achieved by comparing the observed threshold cyclic (Ct) of the sample to the Ct values of the standard curve with known concentrations of human DNA, Promega (Catalogue number: G3041/G304A).

### Data Analysis

Skin and blood parasite loads were summarized by median and interquartile range (IQR). Proportions of positive samples were compared using a chi-square test. Results were summarized per disease status and per skin sampling location (arm, neck, and lesion). Parasite burden in skin samples per disease category were graphically represented using boxplots. Differences in parasite load between different disease groups were assessed using the Mann-Whitney U test. The correlation between skin parasite load and blood parasite load was graphically represented by scatter plots and quantified by (non-parametric) Kendall tau’s correlation coefficient, using the average skin parasite load (of all two or three available skin samples per individual). For the calculation of the chance corrected agreement (kappa index) between presence of parasite DNA in skin and blood, a person was considered positive for skin parasites if at least one skin sample was positive. The correlation between the parasite load of skin samples from different skin sampling sites was presented by scatter plots and Kendall tau’s correlation coefficient, and chance corrected agreement was calculated using kappa index. Jitter method, allowing minimal freedom to the results, was used in all scatter plots, in order to optimize visualization of overlapping points.

### Ethical Considerations

Ethical approval for all study procedures was obtained both in Belgium (from the Institutional Review Board of the Institute of Tropical Medicine, Antwerp, and from the Ethics Committee of the Antwerp University hospital) and in India (from the Institutional Review Board of Banaras Hindu University). Informed consent was obtained from all participants before inclusion. Children (<18 years) were excluded from this study.

## Results

### Participant Characteristics

A total of 201 participants were included in this study; 29 VL patients, 28 PKDL patients, 94 asymptomatically infected individuals, 22 endemic controls, and 28 non-endemic controls. Median age of participants was 36 years (IQR 26–48), with 58% (116/201) of participants being male. Median age was significantly higher among asymptomatically infected individuals and significantly lower in non-endemic controls compared to the other groups. Sixty-one percent (17/28) of PKDL patients had nodular PKDL, 39% (11/28) had the macular form. Main characteristics of participants are shown in **Table 1**.

**Table 1:** Summary of demographic characteristics and blood test results of the included participants per disease category.

	* *	VL	PKDL	Asymptomatics	EC	NEC
**Demographic characteristics**						
	*Prop. Males*	59% (17/29)	75% (21/28)	41% (39/94)	50% (11/22)	100% (28/28)
	*Median age (years)* *(IQR)*	35 (21–45)	33 (30–45)	40 (30–55)	32 (24–46)	28 (22–34)
**Blood results**						
	*DAT positive (%)*	–	–	69% (65/94)	0% (0/22)	0% (0/28)
	*ELISA positive (%)*	–	–	88% (83/94)	0% (0/22)	0% (0/28)
	*WBA (%)*	69% (20/29)	61% (14/23)	53% (50/94)	0% (0/22)	0% (0/28)
	*Blood qPCR positive (%)*	76% (22/29)	0% (0/28)	0% (0/88)	0% (0/22)	0% (0/28)
	*Median blood parasite load in PGE/ml (IQR)*	594.00 (154.0–4,180.00)	<3.75 (0.00 –<3.75)	0.00 (0.00–0.00)	0.00 (0.00–0.00)	0.00 (0.00–0.00)
**Total participants**		29	28	94	22	28

PGE, Parasite Genome Equivalent; IQR, interquartile range; EC, endemic controls; NEC, non-endemic controls.

### Correlation Between Skin Parasite Load and Disease Category

**Figure 1:**
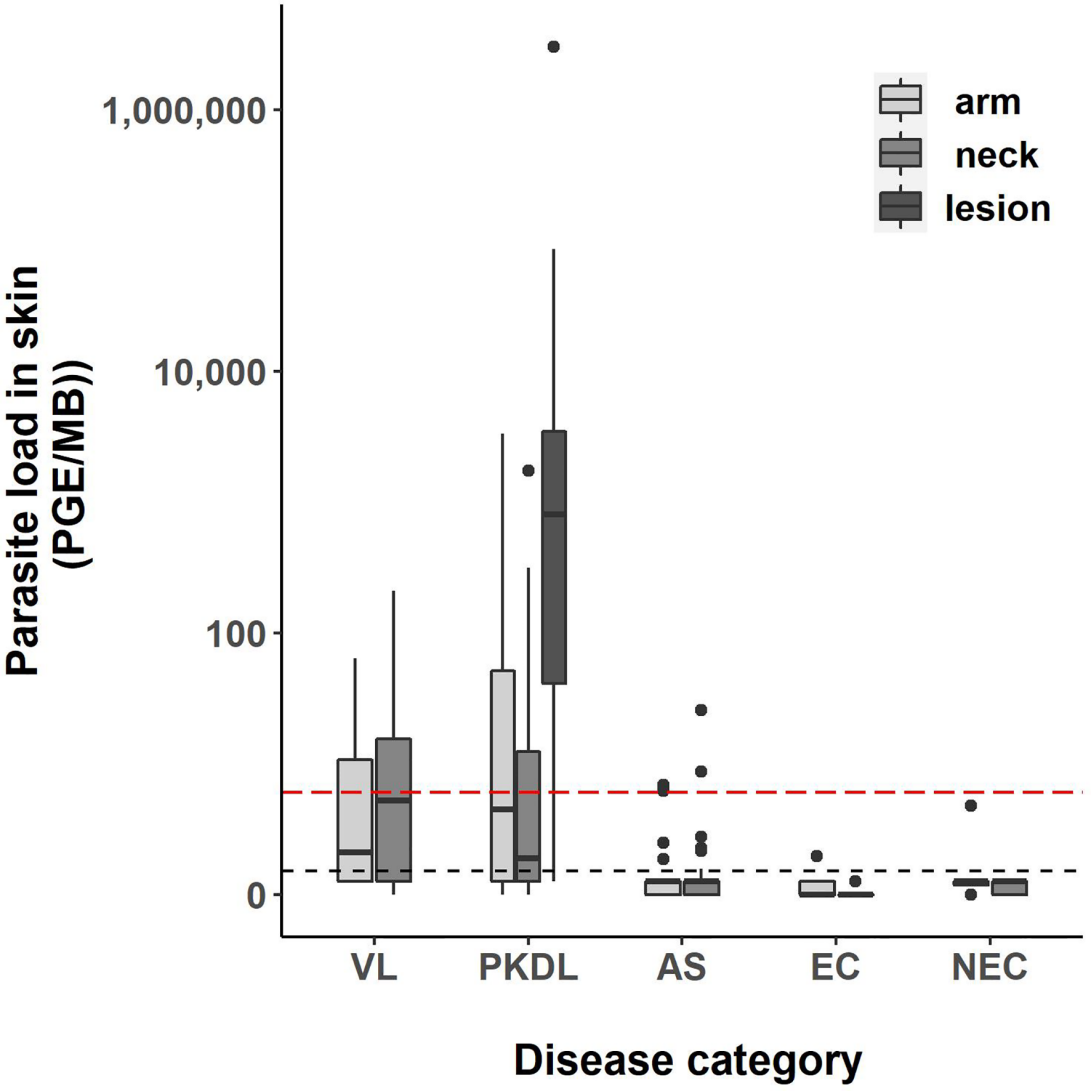
Boxplot of the parasite load of each skin sample [log10(PGE/MB+1)] per disease status. The body of the boxplot represents the median and IQR; whiskers reach 1.5× IQR. PGE = Parasite Genome Equivalent. MB, microbiopsy device; IQR, interquartile range; AS, asymptomatic individuals. The red dotted line represents the cut-off value to identify positive skin samples. The red dotted line defines the lower limit of the linear dynamic range (values above zero but below this line cannot be quantified).

Four skin samples in which no human DNA was found were excluded from analysis as this suggested that no DNA was isolated. A total of 422 skin samples were included for analysis; 197 arm samples, 199 neck samples, and 26 samples from a PKDL lesion. Overall, the skin parasite load found in VL and PKDL patients was significantly higher than in asymptomatics, EC and NEC. **Figure 1** illustrates the skin parasite load found per sampling site and disease status. The mean skin parasite load per disease status and the skin parasite load of the sample with the highest parasite load per disease status can be found in **Supplementary Figures 1** and **2**. 55% of VL patients and 86% of PKDL patients had at least one positive skin sample, while none of the endemic or non-endemic controls had any skin sample positive (**Table 2**). Interestingly, four skin samples from three different asymptomatic individuals were positive. These individuals were contacted two years after sampling had been carried out, but had not developed symptoms of VL by then. Characteristics of these three asymptomatic individuals are provided in **Supplementary Table 1**. The parasite load on the PKDL lesion was significantly higher in patients with nodular PKDL (median 1645 PGE/MB, IQR 667–8348 PGE/MB) than in those with macular PKDL (median 26 PGE/MB, IQR 6–56 PGE/MB), but no such difference was found between arm and neck samples.

**Table 2:** Summary results of skin samples obtained per disease category.

	VL	PKDL	Asymptomatics	EC	NEC
**Skin results**					
**Median (IQR)**					
*Parasite load in arm samples*	1.08 (<0.5–9.75)	3.48 (<0.5–50.33)	<0.5 (0.00–<0.5)	0.00 (0.00–<0.5)	<0.5 (<0.5–<0.5)
*Parasite load in neck samples*	4.23 (<0.5–14.50)	0.89 (<0.5–11.50)	<0.5 (0.00–<0.5)	0.00 (0.00–0.00)	<0.5 (0.00–<0.5)
*Parasite load in lesion samples*	–	803.50 (39.78–3595.00)	–	–	–
*Parasite load of skin sample with highest parasite load*	5.98 (1.14–23.40)	566.50 (32.50–2545.00)	<0.5 (<0.5–<0.5)	0.00 (0.00–<0.5)	<0.5 (<0.5–<0.5)
*Mean parasite load of all samples per individual combined*	4.13 (0.70–13.28)	188.96 (11.33–1159.64)	<0.5 (<0.5–<0.5)	0.00 (0.00–0.00)	<0.5 (<0.5–<0.5)
**Proportion positive (%) (n)**					
*Arm*	30% (8/27)	39% (11/28)	2% (2/92)	0% (0/22)	0% (0/28)
*Neck*	49% (13/29)	36% (10/28)	2% (2/93)	0% (0/21)	0% (0/28)
*Lesion*	–	85% (22/26)	–	–	–
*At least one overall skin sample*	55% (16/29)	86% (24/28)	3% (3/94)	0% (0/22)	0% (0/28)
*Mean parasite load*	45% (13/29)	82% (23/28)	1% (1/94)	0% (0/22)	0% (0/28)
**Total participants**	29	28	94	22	28

PGE, Parasite Genome Equivalents; MB, microbiopsy device; IQR, interquartile range; EC, endemic controls; NEC, non-endemic controls.

### Correlation Between Skin Parasite Load and Blood Parasite Load

**Figure 2:**
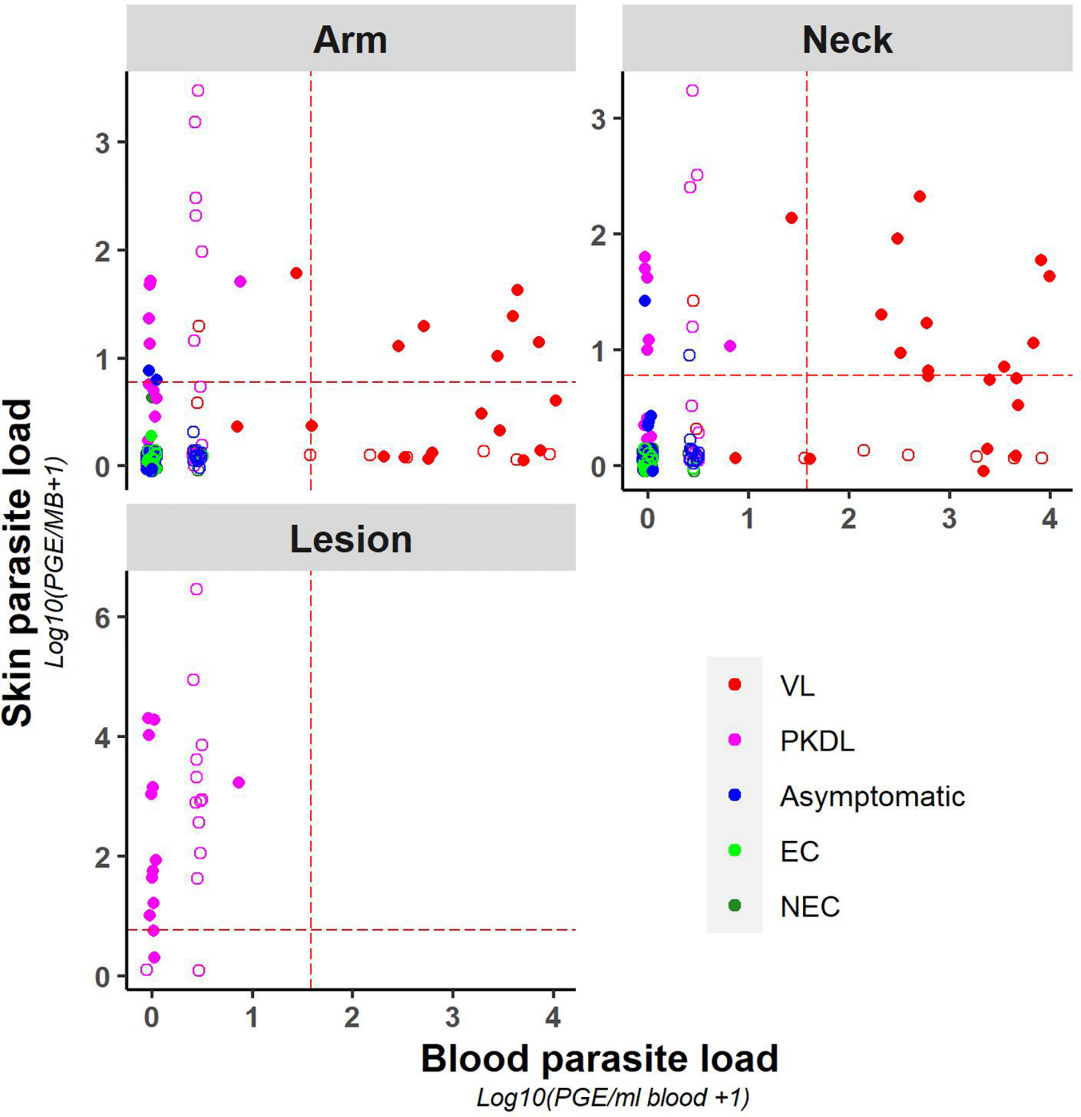
Scatter plot of skin parasite load (log10(PGE/sample + 1)) versus blood parasite load (log10(PGE/ml blood + 1)) per sampling site. The red dotted line represents the cut-off value used to identify positive samples. Empty dots represent values that were not quantifiable (outside of the linear dynamic range of the standard curve).

A weak positive correlation was found between blood and (mean) skin parasite load, with a correlation coefficient of 0.41 (**Supplementary Figure 3**) (graphical presentation of correlation using the skin sample with the highest skin parasite load can be found in **Supplementary Figure 4**). **Figure 2** illustrates the correlation between blood and skin parasite load for the different sampling sites on the skin. The correlation between blood and skin parasite load was higher for arm and neck samples than for lesion samples (Kendall tau τ = 0.38, 0.36, and 0.23 respectively).

Similarly, chance corrected agreement between blood and skin parasite load was low, with a kappa index of 0.30 (p<0.001) as illustrated in **Table 3**. Agreement between blood and skin was higher for neck samples (k=0.40; p<0.001) than for arm samples (k = 0.21; p = 0.004), though remained low for both. No agreement was found between blood and lesion samples (all blood samples from PKDL patients were negative).

**Table 3:** Agreement between a positive skin result and its associated blood sample (Kappa index = 0.30 (p<0.001)).

	Skin positive - n(%)	Skin negative - n(%)
Blood positive - n(%)	13 (7%)	9 (5%)
Blood negative - n(%)	30 (15%)	143 (73%)

### Correlation Between Different Skin Samples

**Figure 3:**
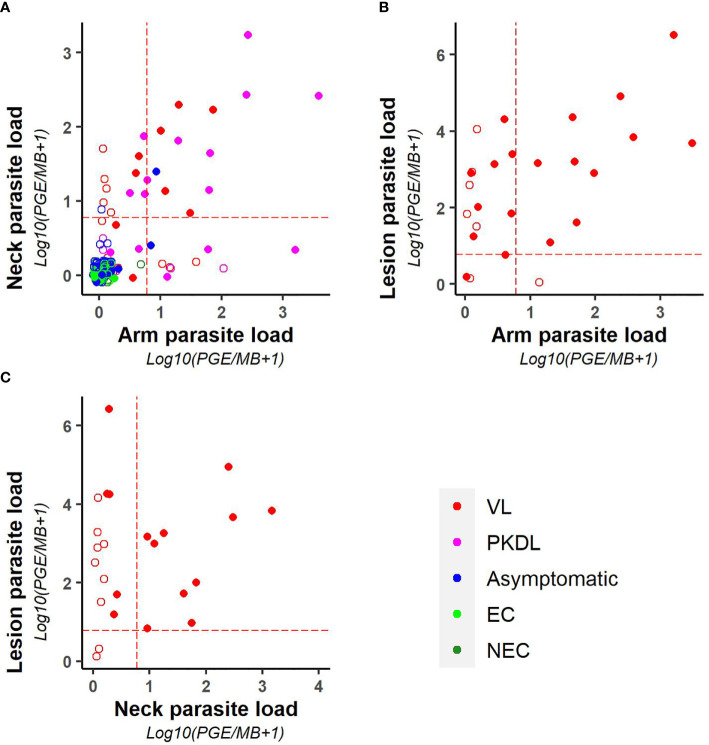
Scatter plots illustrating the correlation between the parasite loads of **(A)** arm and neck, **(B)** arm and lesion, and **(C)** neck and lesion samples. Parasite loads are expressed as log10(PGE/sample +1). The red dotted line represents the cut-off value used to identify positive samples. Empty dots represent values that were not quantifiable (outside of the linear dynamic range of the standard curve).

As illustrated in **Figure 3**, parasite loads in arm and neck samples were moderately correlated (Kendall tau τ = 0.51; p-value <0.001), with similar proportions of positive results in both (11.2% (22/197) versus 12.6% (25/199) respectively). Also on a binary scale (positive versus negative), chance corrected agreement between arm and neck samples was moderate (kappa = 0.47, p<0.001) (**Table 4**). The parasite load found in PKDL lesions was significantly higher than the parasite load found in arm and neck samples, with 85% (22/26) of lesion samples testing positive. Correlation between the parasite load in arm or neck samples and lesion samples was weak (= 0.36 (p = 0.01) and 0.24 (p = 0.11) respectively).

**Table 4:** Agreement between skin samples from different sampling sites.

	Arm + n(%)	Arm - n(%)		Arm + n(%)	Arm - n(%)		Neck + n(%)	Neck - n(%)
**Neck +**	12 (6%)	12 (6%)	**Lesion +**	10 (38%)	12 (46%)	**Lesion +**	9 (35%)	13 (50%)
**Neck –**	9 (5%)	162 (83%)	**Lesion –**	1 (4%)	3 (12%)	**Lesion –**	1 (4%)	3 (12%)
	*kappa = 0.47 (p<0.001)*			*kappa = 0.10 (p=0.45)*			*kappa = 0.07 (p=0.55)*	

## Discussion

In this proof of concept study, we found a clear correlation between the skin parasite load obtained with the microbiopsy device and disease status, with both higher skin parasite loads and a higher proportion of positive skin samples in VL and PKDL patients compared to asymptomatics, EC, and NEC. No clear correlation between skin parasite load and blood parasite load was found, but a moderate correlation was present between the skin parasite load in arm and neck samples. In line with previous pilot studies, our results from an Indian setting suggest that the microbiopsy device provides a promising tool to measure skin parasite load in the Indian setting, and – if validated by xenodiagnosis studies – could facilitate much needed larger scale studies on infectiousness of human subgroups.

The main strength of this study is that it is the first study to our knowledge that pilots the use of the microbiopsy device in the setting of the Indian subcontinent, and only the third to pilot this on different disease/infection subgroups for leishmaniasis worldwide (Kirstein et al., 2017; Churiso et al., 2020). An additional strength of this study lies in the fact that four different tests (DAT, rK39 ELISA, WBA, and blood parasite load) were used to identify asymptomatically infected individuals. Asymptomatic infection is not uniformly defined, with different studies using different markers to identify infection. As different markers might point to different subgroups of infection—e.g. early versus late infection, etc.—we attempted to identify a broad spectrum of asymptomatically infected individuals by combining several tests.

This study had several limitations. First, the relatively small sample size of this pilot study was chosen based on feasibility within the limited time frame rather than based on calculations. Secondly, non-endemic controls were selected among students attending Banaras Hindu University, Varanasi, which is based within the VL endemic state of Uttar Pradesh, although the city of Varanasi itself is assumed to be non-endemic for VL. As students originate from different states across India, it cannot be excluded that some originate from an area where VL is endemic, and should therefore have been categorized as endemic controls rather than non-endemic controls.

We used the microbiopsy device as described by Lin et al. (2013). This device was used for the first time in the context of leishmaniasis by Kirstein et al., 2017 (Kirstein et al., 2017), who found positive skin samples in all VL, PKDL and CL patients (total n = 10), as well as in almost half (48.4% = 121/250) of the asymptomatically infected individuals. Several factors may have contributed to the higher proportions of skin positives among the different groups in the Kirstein et al. study compared to ours. First, the cut-off used by Kirstein et al. to define positivity in the skin was much lower; 1 PGE/ml (corresponding to +– 0.003PGE/device) versus 1 PGE/PCR (corresponding to 5 PGE/device) in our study. Our choice for a cut-off titer was based on the highest parasite load found in negative (no-template) controls (cfr paragraph on laboratory analyses). Secondly, a large group of asymptomatically infected individuals in Kirstein’s study were recovered VL cases, which might reflect a higher chance of lingering parasite DNA in both blood and skin.

At present, the most widely accepted alternative for xenodiagnosis as a marker for infectiousness is blood parasite load, usually measured by qPCR (Miller et al., 2014). A recent study, however, suggested only a moderate correlation between blood parasite load measured by microscopy and by qPCR, highlighting the fact that molecular methods capture mainly naked circulating DNA attributed to recently dead parasites from other tissues (Silva et al., 2016). Several studies found that skin parasite load on the other hand was actually a better marker for infectiousness than blood parasite load (Courtenay et al., 2014; Doehl et al., 2017). This hypothesis is supported by several authors stating that the parasite load measured by microscopy in the blood is too low to account for the findings in xenodiagnosis studies, while the parasite load in the skin is much higher, suggesting skin and not blood to be the source of infection (Silva et al., 2016; Doehl et al., 2017). An important hurdle to prove this hypothesis, however, is the fact that parasite load in the skin is not a uniformly defined parameter, but is rather heterogeneously distributed throughout the skin (Doehl et al., 2017). This patchy distribution of parasites in the skin might reduce the number of sand flies taking up parasites, but also increases the parasite load in those feeding on a patch, optimizing their potential for onward transmission, which might be of particular importance in individuals with low overall parasite loads, such as asymptomatic individuals (Doehl et al., 2017). Patchiness of parasites in the skin has been linked to persisting parasites after an infectious bite. As also documented for other vector-borne diseases such as malaria and dengue (Frischknecht, 2007; Ménard et al., 2013), Aslan et al. (2016) illustrated that *L. infantum* parasites were accessible to sand flies at the site of an infectious bite in dogs up to one year later. If sand flies would demonstrate preferred biting sites in humans – as is the case in several animals – this could mean an important advantage towards transmission potential, although this has yet to be established. This heterogeneity most likely also explains why we only found a moderate correlation between the skin parasite loads in arm and neck samples, with only half of the individuals who presented with a positive (arm or neck) sample providing also a second positive (arm or neck) sample.

While the potential of diseased individuals to transmit *Leishmania* parasites to sand flies is firmly established (Swaminath et al., 1942; Costa et al., 2000; Vergel et al., 2006; Molina et al., 2017; Mondal et al., 2019), human xenodiagnosis studies have never been able to illustrate transmission from asymptomatic individuals - although it needs to be noted that the number of studies on this topic is limited (Costa et al., 2000; Singh et al., 2021). There are, however, some signs pointing in this direction, such as the presence of live *L. donovani* amastigotes in the blood of asymptomatically infected individuals in a VL endemic Indian village (Sharma et al., 2000). In addition, mathematical modeling strongly suggests that asymptomatic infections indeed play a role in maintaining transmission (Stauch et al., 2011). Asymptomatic animals on the other hand have been reported to transmit *Leishmania* parasites. While results from individual studies are sometimes conflicting (Guarga et al., 2000; Travi et al., 2001; Michalsky et al., 2007; da Costa-Val et al., 2007; Verçosa et al., 2008), a meta-analysis on transmission potential of dogs asymptomatically infected with *L. infantum* concluded that asymptomatic animals indeed contribute to transmission, albeit to a lesser extent than symptomatic animals (Quinnell and Courtenay, 2009). While fewer asymptomatic dogs seem to infect sand flies, the proportion of sand flies infected after feeding upon an infectious asymptomatic dog were found to be similar or even higher than for symptomatic dogs (Quinnell and Courtenay, 2009; Laurenti et al., 2013). Similar results were found for transmission of other parasite species in animals, including *L. tropica* and *L. major* (Svobodová et al., 2006; Sadlova et al., 2015).

Interestingly, we found four skin samples from asymptomatically infected individuals taken with the microbiopsy device to be positive with parasite DNA. It must be noted here, however, that presence of parasite DNA does not automatically mean the presence of a whole – potentially infective – parasite. Whenever we mention parasite load, we actually refer to the number of PGE per ml blood or microbiopsy sample. However, PCR can also detect DNA debris, as evidenced by samples containing less than one PGE. Even when a 200 µl blood sample or 2–3 µl microbiopsy does not contain a single parasite, it is thus still possible to detect more than one PGE in it. Studies directly linking the results of this microbiopsy tool to those of xenodiagnosis would be able to shed light on the association between parasite DNA presence in the skin and infectiousness to sand flies.

A study assessing the microbiopsy device as an alternative for slit skin smears for the diagnosis of CL was recently published (Churiso et al., 2020). The authors found the microbiopsy device to outperform routine skin slit sampling in the Ethiopian setting, identifying it as a promising alternative for CL diagnosis. Although no CL lesions were included in our study, 85% (22/26) of PKDL lesions tested positive with the microbiopsy device. While the sensitivity was higher for nodular PKDL (94% = 15/16) than for the macular form (70% = 7/10), this could be further optimized by identifying the most accurate diagnostic cut-off value for this aim. In addition, contrary to the current diagnostic tools such as skin slit smear or skin biopsy, the MB device would have the advantage that samples can more easily be taken in field conditions, requiring no anesthesia nor specialized care – a trait which is especially interesting with the increasing focus on PKDL patients as long-term reservoirs for transmission. We therefore advocate further evaluation of the MB device as a diagnostic tool for PKDL.

## Conclusions and Future Research

In line with previous pilot studies, our results from an Indian setting suggest that the microbiopsy device provides a promising tool to measure skin parasite load, and – if validated by xenodiagnosis studies – could facilitate much needed larger scale studies on infectiousness of human subgroups. In addition, it could provide an alternative diagnostic tool for PKDL lesions, with advantages over the current diagnostic tools for use in field settings.

## Data Availability Statement

The raw data supporting the conclusions of this article will be made available by the authors, without undue reservation.

## Ethics Statement

The studies involving human participants were reviewed and approved by the Institutional Review Board of the Institute of Tropical Medicine, Antwerp, Belgium, the Ethical Committee of the Antwerp University Hospital, Belgium, and the Ethics Committee of Banaras Hindu University, Varanasi, India. The patients/participants provided their written informed consent to participate in this study.

## Author Contributions

MB, OS, EH, and KC contributed to the conception and design of the study. Data curation was performed by OS, GV, AS, PK, MG, and TR. Formal analysis was performed by GV and KC and validated by EH, SS, and MB. Writing of the original draft manuscript was performed by OS and KC. All authors contributed to the article and approved the submitted version.

## Funding

This study was funded by the Pump Priming Project programme from the Institute of Tropical Medicine, Antwerp, supported by the Department of Science, Technology and Innovation of the Flemish government. In addition, it was partly supported by the Extramural Program of the National Institute of Allergy and Infectious Diseases, National Institutes of Health (TMRC grant number U19AI074321).

## Conflict of Interest

The authors declare that the research was conducted in the absence of any commercial or financial relationships that could be construed as a potential conflict of interest.
