# Supplementary material for: Assessing L. donovani Skin Parasite Load: A Proof of Concept Study of a Microbiopsy Device in an Indian Setting
Source: Front Cell Infect Microbiol. 2021 Mar 11;11:645121. doi: 10.3389/fcimb.2021.645121 (PMC8006290; doi:10.3389/fcimb.2021.645121)
Supplement: Supplementary file 1 [file DataSheet_1.pdf]

<u>**SUPPLEMENTARY MATERIALS**</u>

Supplementary Figure 1 *:* Boxplot of the parasite load (log10(PGE/MB+1)) per disease status, using the **mean** of all samples taken from an individual. The body of the boxplot represents the median and IQR; whiskers reach 1.5x IQR. MB = Microbiopsy device. PGE = Parasite Genome Equivalent. IQR = Interquartile Range. The red dotted line represents the cut-off value to identify positive skin samples. The black dotted line defines the lower limit of the linear dynamic range (values above zero but below this line cannot be quantified).

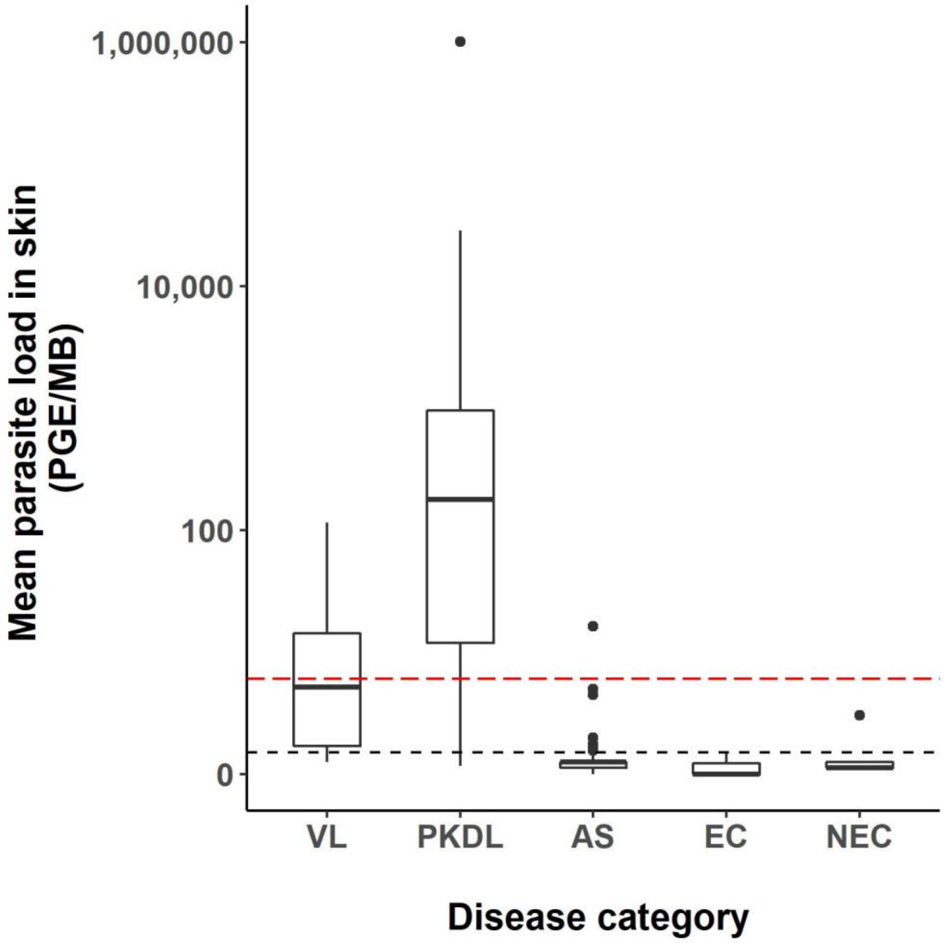

Supplementary Figure 2 : Boxplot of the parasite load (log10(PGE/MB+1)) per disease status, using only the skin sample with the **highest** parasite load. The body of the boxplot represents the median and IQR; whiskers reach 1.5x IQR. MB = Microbiopsy device. PGE = Parasite Genome Equivalent. IQR = Interquartile Range. The red dotted line represents the cut-off value to identify positive skin samples. The black dotted line defines the lower limit of the linear dynamic range (values above zero but below this line cannot be quantified).

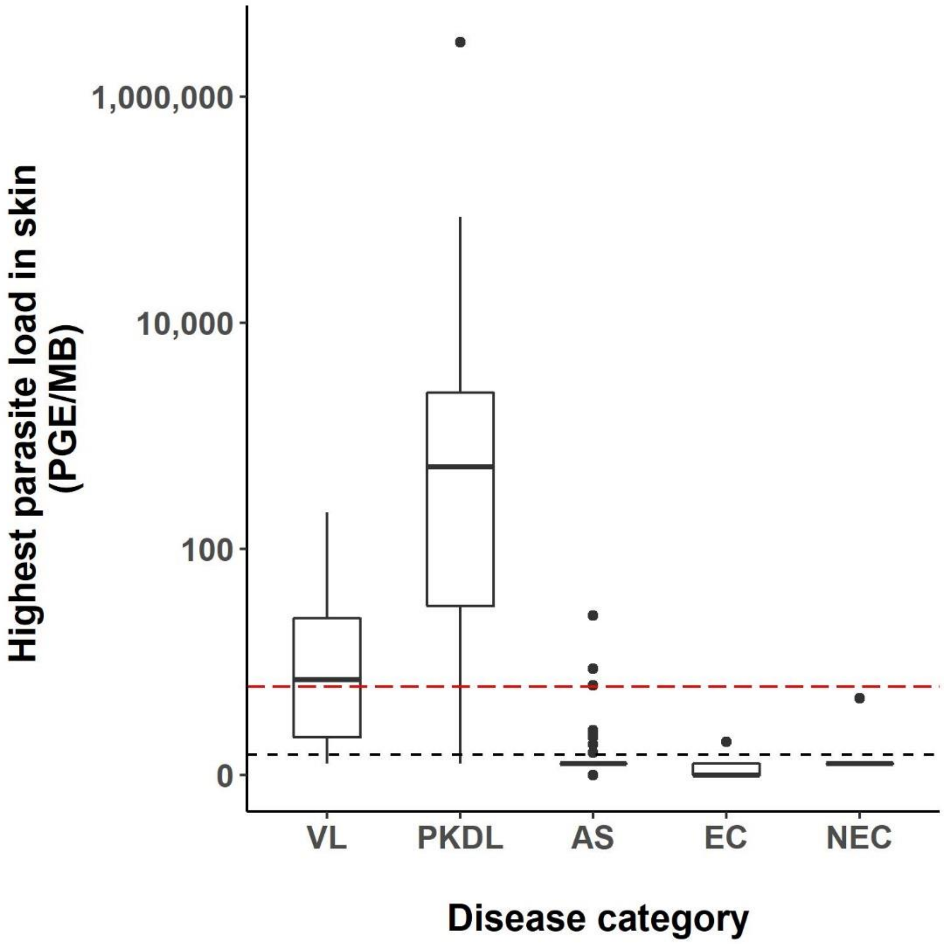

Supplementary Figure 3: Scatter plot of the **mean** skin parasite load (log10(PGE/sample + 1)) versus blood parasite load (log10(PGE/ml blood + 1)) per sampling site. The red dotted line represents the cut-off value used to identify positive samples. The red dotted line represents the cut-off value used to identify positive samples. Empty dots represent values that were not quantifiable (outside of the linear dynamic range of the standard curve).

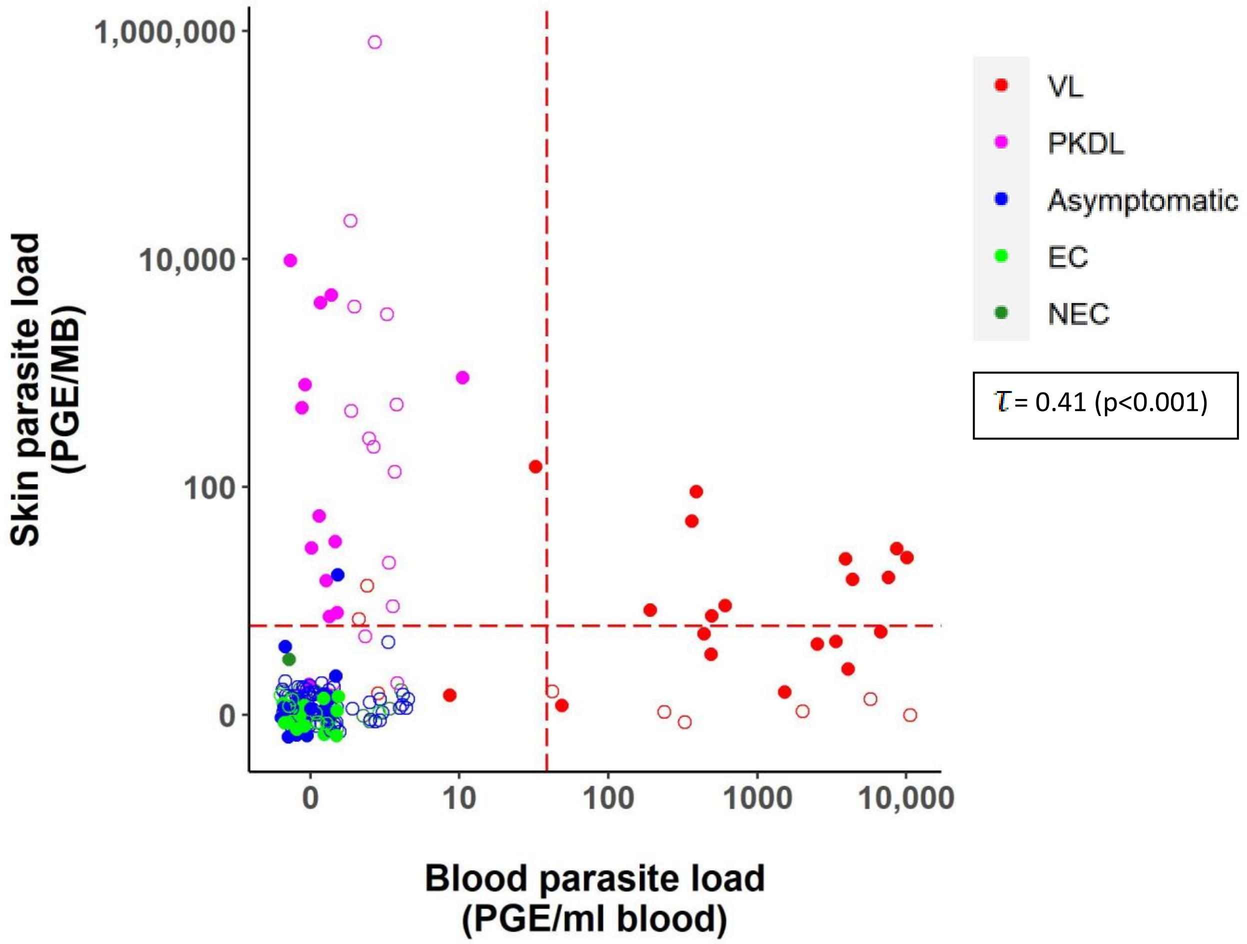

Supplementary Figure 4: Scatter plot of the **highest** skin parasite load (log10(PGE/sample + 1)) versus blood parasite load (log10(PGE/ml blood + 1)) per sampling site. The red dotted line represents the cut-off value used to identify positive samples. The red dotted line represents the cut-off value used to identify positive samples. Empty dots represent values that were not quantifiable (outside of the linear dynamic range of the standard curve).

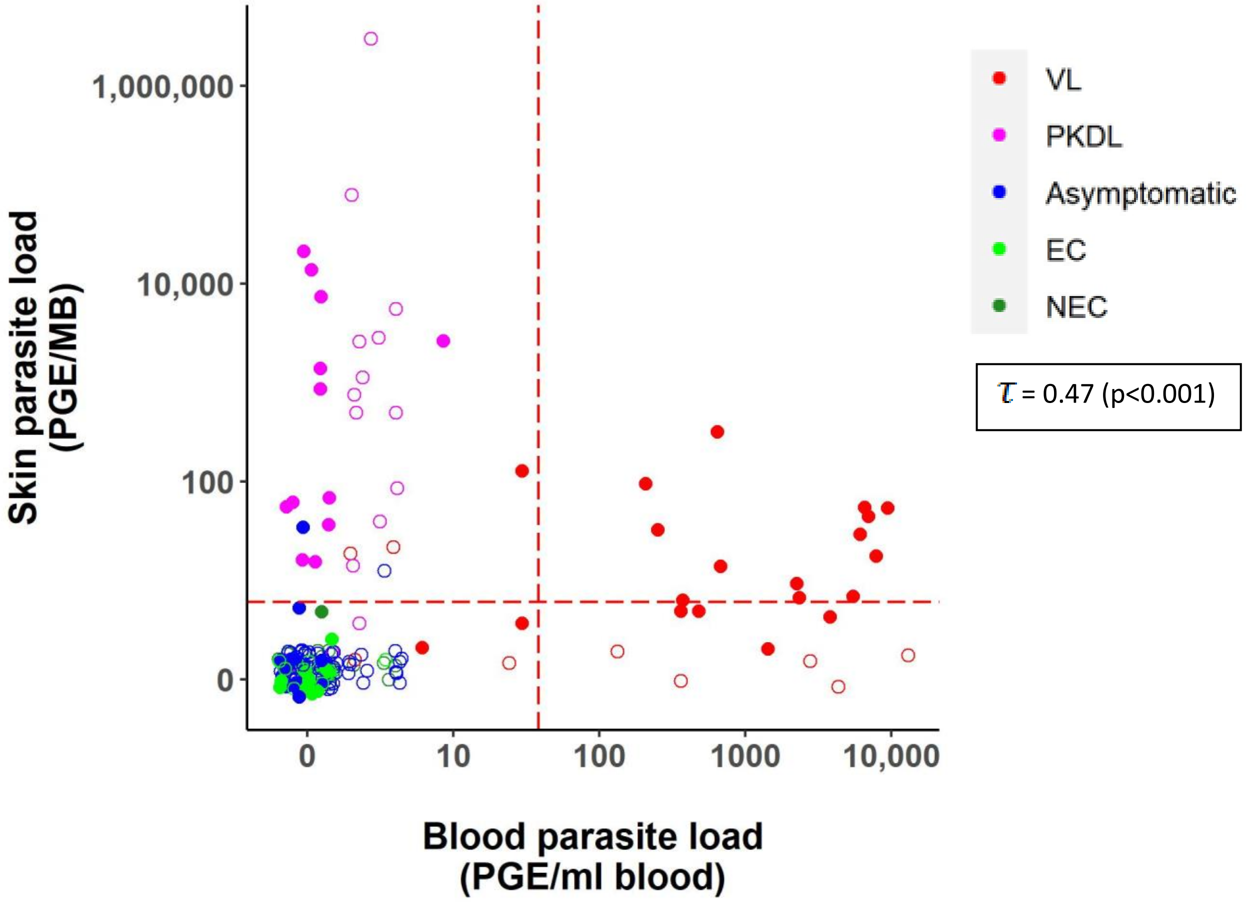

Supplementary Table 1: Characteristics of asymptomatically infected individuals with at least one positive skin sample. Positive results are marked in bold.

Age (years)	Sex	DAT titer	Elisa (PP value)	Quantiferon (pg/ml)	qPCR Blood (PGE/ml)	qPCR Arm (PGE/MB)	qPCR Neck (PGE/MB)
26	Female	**≥ 1:3,200**	**32.73**	**59.47**	negative	**5.85**	**24.60**
60	Female	≥ 1:400	**26.91**	**565.26**	<3.75	<0.50	**7.67**
55	Female	≥ 1:800	**27.92**	36.55	negative	**5.16**	1.76

MB : Microbiopsy device. PGE = Parasite Genome Equivalent. DAT = Direct Agglutination Test. PP = percentage point positivity.
